# The Protective Roles of Dietary Selenium Yeast and Tea Polyphenols on Growth Performance and Ammonia Tolerance of Juvenile Wuchang Bream (*Megalobrama amblycephala*)

**DOI:** 10.3389/fphys.2018.01371

**Published:** 2018-10-02

**Authors:** Honghui Guo, Wang Lin, Jie Hou, Lingkai Wang, Dandan Zhang, Xueyang Wu, Li Li, Dapeng Li

**Affiliations:** ^1^College of Fisheries, Huazhong Agricultural University, Wuhan, China; ^2^Hubei Provincial Engineering Laboratory for Pond Aquaculture, Wuhan, China; ^3^National Demonstration Center for Experimental Aquaculture Education – Huazhong Agricultural University, Wuhan, China

**Keywords:** ammonia stress, selenium yeast, tea polyphenols, gene expression, ultra-pathology, Wuchang bream

## Abstract

In order to investigate protective roles of dietary selenium yeast (SY) and tea polyphenols (TPs) on growth of juvenile Wuchang bream (*Megalobrama amblycephala*) and its resistance under ammonia stress, juvenile Wuchang bream were randomly assigned into four groups: a control group fed basal diets and three treatment groups fed basal diets supplemented with 0.50 mg/kg SY, 50 mg/kg TPs and a combination of 0.50 mg/kg SY and 50 mg/kg TPs, respectively. After 60 days of feeding, growth parameters of Wuchang bream were measured along with serum hormones and the transcription of growth axis-related genes. Then fish were exposed to ammonia stress of 22.5 mg/L total ammonia nitrogen. Hepatic oxidative damage parameters, antioxidant responses and ultrastructure were evaluated before ammonia exposure (0 h) and at 3, 6, 12, 24, and 48 h after ammonia exposure. Results show that before ammonia exposure, the growth parameters, serum GH and IGF-1 levels as well as the growth axis-related gene expression (*gh, ghr2* and *igf-1*) for the SY and combination groups were higher than those determined for the fish on the control diet. In contrast, the administration of TP alone didn’t have significant effects on the growth parameters and growth-related hormones. After ammonia exposure, compared with the control, remarkable increases in the activity and mRNA expression of hepatic antioxidant enzymes (glutathione peroxidase and catalase superoxide dismutase) in three treatment groups were observed along with decreases of hepatic malondialdehyde and protein carbonylation levels, indicating that the single and combined supplementation of SY and TPs could enhance antioxidant capacity to alleviate oxidative stress and damage by ammonia. Consistent with this finding, alterations of the liver ultrastructure in three treatment groups were less severe and faster recovery than in the control group after ammonia exposure. In conclusion, a basal diet supplemented with the combination of 0.50 mg/kg SY and 50 mg/kg TPs could has very beneficial effects on the whole aspects of the growth and ammonia resistance in Wuchang bream juveniles.

## Introduction

Ammonia is one of the major pollutants in aquatic environment. It is a product of ammonification and also a nitrogenous excretory product of bony fish (teleosta) (Alcaraz et al., 1997). Under intensive farming condition, ammonia concentrations may easy rise to unsafe levels, which leads to poor growth, lower immunity, higher susceptibility to bacterial pathogens, and thus high mortality in fish (Reddy-Lopata et al., 2006; Sun et al., 2012; Schram et al., 2014). High levels of ammonia currently became a crucial restricting factor in intensive aquaculture. Recent studies further confirmed that ammonia could induce reactive oxygen species (ROS) production in aquatic animal body and result in oxidative stress and damage (Ching et al., 2009; Sun et al., 2011; Kim et al., 2015; Sinha et al., 2015). Indeed, fish have developed an antioxidant defense system which can neutralize and subsequently scavenge ROS in the cell. The defense system consists of antioxidant enzymes such as superoxide dismutase (SOD), catalase (CAT) as well as glutathione peroxidase (GPx), and non-enzymatic antioxidant molecules like glutathione and vitamin C/E (Wilhelm Filho, 1996; Kelly et al., 1998). However, cell oxidative stress is triggered when pro-oxidant forces exceed antioxidant defense capacity, and this situation will damage proteins and lipids in the cellular compartments (Droge, 2002). Aiming at this problem, antioxidant additive in fish diets is a better way to improve the growth performance, stress responses, and disease resistance from the perspective of animal itself in comparison to antibiotic supplement (Prieto et al., 2008; Ming et al., 2012).

Selenium, an essential trace element, has been proven to improve growth and antioxidant performance in fish diets (Lin and Shiau, 2005; Dörr et al., 2008). Several studies have documented that the main chemical form of organic selenium, selenomethionine is more readily absorbed and potent in the field of bioavailability and health effects than inorganic selenite in fish stuff (Schrauzer, 2003). Due to containing high levels of selenomethionine, selenium yeast (SY) is widely used as a nutritive antioxidant in terrestrial livestock feedstuffs (Schrauzer, 2006). Recently, a few researches have demonstrated that SY supplementation can enhance growth, feed conversion and immunocompetence in fishes (Wang and Lovell, 1997; Cotter et al., 2008; Rider et al., 2009; Nugroho and Fotedar, 2013). As another natural antioxidant, tea polyphenols (TPs) of the main components of green tea are well known with their many functions including antioxidant, antimicrobial, antimutagenic and anticlastogenic, and anticarcinogenic properties in animals (Sakanaka et al., 2000; Gupta et al., 2002; Yokozawa et al., 2002; Duong et al., 2016). Until now, there are very limited information on the application of TP as a potential dietary additive in fish feed and its effects. Nootash et al. (2013) showed that green tea at 100 mg/kg feed induced expression of immune relevant genes and enhanced serum SOD activity in rainbow trout (*Oncorhynchus mykiss*). Green tea was also observed to improve weight gain of the channel catfish (*Ictalurus punctatus*) (Zhou et al., 2015). Song et al. (2015) indicated that green TPs could help recover reproductive functions, egg quality and reduce deformation ratio in medaka (*Oryzias latipes*) induced by benzopyrene. Apparently, all the previous studies were fragment and limited. It is therefore imperative to assess the protective roles of the single or combined use of SY and TP as the potent dietary additive in fish feed and the possible mechanism behind these benefits.

Wuchang bream (*Megalobrama amblycephala*), as one of principal species in Chinese freshwater intensive systems, is suffering from environmental stresses caused by overstocking and water pollution like excessive ammonia. At present, development of an environmental protection feed to promote optimum growth and maintain health status of fish becomes utmost importance in health aquaculture. In light of above, the present study aims to investigate effects of the single and combined use of SY and TP as natural antioxidant supplements in fish feed on growth performance by detecting hormones levels and growth-axis gene expression, and on ammonia resistance through determining the changes in hepatic ultrastructure and antioxidant enzymatic activity as well as transcription of Wuchang bream juveniles exposed to ammonia. The current results would provide new insights for the stress alleviation and disease prevention in fish culture.

## Materials and Methods

### Fish and Experimental Diets

Healthy Wuchang bream juveniles were obtained from Tuanfeng fish farm (Hubei, China). Prior to the experiment, fishes were acclimated in a re-circulating system with continuous aeration for 14 days. During this period, fishes were fed two times daily with a basal diet to near satiation. Ingredients and chemical composition of the basal diet are shown in **Table 1**.

**Table 1:** Ingredients and composition of the basal diet.

Ingredients (%)		Chemical composition (in dry matter)	
White fish meal	15.00	Crude protein (%)	35.09
Soybean meal	49.05		
Fish oil	7.05	Crude lipid (%)	5.48
Corn starch	10.30		
Choline chloride (50%)	1.00	Water (%)	7.18
Carboxyl methyl cellulose	3.00	Ash (%)	14.33
Cellulose	8.60		
Cr_2_O_3_	0.50	Gross energy (KJ/g)	18.04
Ca(H_2_PO_4_)_2_	2.50		
Vitamin premix^1^	1.00		
Mineral premix (no se)^2^	2.00		

*^1^Vitamin premix (mg/kg): riboflavin, 20; thiamin, 20; pyridoxine, 20; vitamin B_12_, 2; vitamin D, 0.5; vitamin A, 1.83; vitamin K, 10; vitamin E, 10; folic acid, 5; Ca-pantothenate, 50; inositol, 100. ^2^Mineral premix (g/kg): NaCl, 0.8; NaH_2_PO_4_⋅7H_2_O, 20; MgSO_4_⋅7H_2_O, 12; KH_2_PO_4_, 25.6; Ca(H_2_PO_4_)_2_⋅H_2_O, 16; (CH_2_CHCOO)_2_Ca⋅5H_2_O, 2.8; FeSO_4_, 2; ZnSO_4_⋅7H_2_O, 0.028; MnSO_4_⋅4H_2_O, 0.013; CuSO_4_⋅5H_2_O, 0.0025; CoCl_2_⋅6H_2_O, 0.0008; KIO_3_⋅6H_2_O, 0.0024.*

In the present experiment, SY (containing 2,000 mg Se/kg) was provided by Angel yeast Co., Ltd., and TP with a purity of ≥95% was obtained from Hangzhou Gosun Biotechnologies Co. Ltd., China. The basal diet was as control group diet, in which the effective content of Se was 0.08 mg/kg assayed by inductively coupled plasma atomic emission spectrometry. And three experimental diets with supplements for the SY group, TP group and combination group of SY and TP were formulated to contain 0.5 mg SY/kg, 50 mg TP/kg and a combination of 0.5 mg SY/kg and 50 mg TP/kg, respectively. The levels of dietary SY and TP used in our study were based on previous researches (Xu et al., 2008; Rider et al., 2009; Nugroho and Fotedar, 2013) and our preliminary results of Wuchang bream fed with graded levels of SY and TP (Long et al., 2015). The diets were manufactured by extrusion machine with 2 mm pellet sizes, air-dried and then stored at −20°C until use.

### Feeding Trial

After an adaptation period, a total of 600 fishes were selected and randomly divided into 12 fiberglass tanks (cylindrical, volume 300 L) at a density of 50 fish. Twelve tanks were randomly assigned for four experimental groups and each group has three replicates. Fish were fed four experimental diets to apparent satiation twice daily (9:00 and 16:00) for 60 days. During the experiment, water temperature was maintained at 25.8 ± 0.2°C, pH was kept at 7.6 and dissolved oxygen was >6.0 mg/L. The photoperiod was 12 h light/12 h dark. Fish were weighted at the beginning and end of the feeding trial.

### Ammonia Stress Test

At the end of feeding trial, fish were starved for 24 h and then used for a short-term exposure test of ammonia. 50 fish from each tank were reserved and exposed to 22.5 mg/L total ammonia nitrogen (TA-N) (50% 48-h LC_50_) for 48 h. The desired concentration was obtained from stock solution made with reagent grade ammonia chloride power (NH_4_Cl, BASFR, 99.5% purity). During the exposure period, concentrations of TA-N were determined once daily by the method of salicylate-hypochlorite (Verdouw et al., 1978). According to the study of Emerson et al. (1975), the concentration of unionized ammonia based on temperature and pH conditions was about 0.55 ± 0.01 mg/L. There was no food supplied to fish during ammonia exposure.

### Sampling and Processing

Before (0 h) and after 3, 6, 12, 24, and 48 h of ammonia exposure, 25 individuals were randomly sampled from each experimental group and anesthetized with tricanine methanesulfonate (MS-222) solution. Bloods were firstly collected to separate the serum for subsequent hormone measurement. In each experimental group, three fish’s liver were fixed 2.5% glutaraldehyde solution for ultrastructure examination. And brain and liver samples of the other fish were excised, frozen immediately with liquid nitrogen and then stored at −80°C for the analysis of gene expression and antioxidant parameters. All animals were handled according to the guidelines of “Guidelines for Experimental Animals” of the Ministry of Science and Technology (Beijing, China), meanwhile the protocol of our study had approved by the Institutional Animal Care and Use Committee (IACUC) of Huazhong Agricultural University, China.

### Serum Hormone Assay

Blood samples were centrifuged for 30 min (845 × *g*, 4°C) and then the serum were separated immediately. One replication of serum samples contains five fishes’ serum and were stored at −80°C until analysis. Five replicates were used in each treatment. Growth hormone (GH) and insulin-like growth factor 1 (IGF-1) in serum were determined, respectively, using commercial ELISA kits of the Nanjing Jiancheng Bioengineering Co. (Nanjing, China) according to manufacturer’s instructions. In brief, the kits were to use the competitive assays based on monoclonal antibodies to GH and IGF-1 of zebrafish. Purified zebrafish GH and IGF-1 were, respectively, used as standards (ng/ml). Fifty-microliters of serum samples or standards, diluted serially with PBS-BSA was added to each well which was pre-coated with monoclonal antibodies, and then added 50 μL of horseradish peroxidase-labeled-antigens for immunoprecipitation of bound fraction. After incubation for 1 h at 37°C, the plate was washed five times with PBS-0.1% Tween buffer (PBST). Then enzymatic color reaction was developed at room temperature in the dark by adding 100 μL of TMB-peroxide substrate solution to each well. The reaction was stopped by adding 50 μL of 2 M H_2_SO_4_ per well, and the absorbance at 450 nm was measured using a microplate reader (Bio-Rad 680, United States). The reliability of the heterologous immunoassay for the kits was further analyzed by parallel dilution curve between zebrafish standard and Wuchang bream serum as described in Marchelidon et al. (1996) and Dyer et al. (2004). The serial dilutions of bream serum were parallel to zebrafish standards (**Supplementary Figure 1**). Therefore, the obtained relative changes in GH and IGF-1 contents were correct although the obtained values were likely different from the exact absolute values for Wuchang bream GH and IGF-1. The intra-assay coefficients of variations for the kits of GH and IGF-1 were all lower than 10%, while their inter-assay variations were lower than 12%. The sensitivity of kits was 0.1 ng/mL and 3 ng/mL, respectively, for GH and IGF-1 as well as their recoveries were 95% and 88%.

### Measurement of LPO, Protein Carbonylation, and Antioxidant Parameters

Liver samples from three fishes were pooled for one replicate and homogenized in ice-cold 0.85% NaCl (1:10 w/v). Homogenates were centrifuged for 10 min (845 × *g*, 4°C) and supernatants were separated for detecting antioxidant parameters. The levels of lipid peroxidation (LPO) and protein carbonylation (PC) as well as activities of CAT and GPx were measured by the commercial kits of Nanjing Jiancheng Bioengineering Co. (Nanjing, China). The concentration of malondialdehyde (MDA), as an index of LPO, was detected by the method of Ohkawa et al. (1979). PC contents were obtained by detecting the formation of protein hydrazones using 2,4-dinitrophenylhydrazine (Armenteros et al., 2009). CAT activity was detected though measuring the rate of disappearance of H_2_O_2_ by slight modifications (Aebi, 1984). GPx activity was obtained though determining coupling of GSH and 5,5′-dithiobis-(2-nitrobenzoic)-acid (DTNB) (Drotar et al., 1985). Protein concentration was detected by the Coomassie blue method using bovine serum albumin as a standard (Bradford, 1976).

### Gene Expression Analysis

Total RNA was extracted from brain and liver samples using the TRIzol reagent (TaKaRa, Dalian, China) according to the manufacturer’s instructions. The quality and integrity of RNA were tested by the NanoDrop ND-2000 (Thermo Scientific, Wilmington, DE, United States) and evaluated by 1% agarose gel electrophoresis. When the A_260_/A_280_ values were in the range of 1.8–2.0 and A_260_/A_230_ values were above 2.0, 1 μg of total RNA from each sample were used for reverse transcription using PrimeScript^TM^ RT reagent Kit with gDNA Eraser (Takara, Dalian, China). Quantitative real-time PCR (qPCR) assay was performed on a Roche Light Cycler 480 machine (Roche, Sussex, United Kingdom) with iQ^TM^ SYBR^®^ Green Supermix (Bio-Rad Laboratories, Hercules, CA, United States), containing 10 μl iQ SYBR^®^ Green Supermix, 0.8 μl of each of the specific primers, 6.4 μl of RNase-free water and 2 μl of cDNA template. The thermal program included 10 min at 94°C, 40 cycles at 94°C for 10 s, 60°C for 20 s, and 72°C for 30 s. The gene-specific primers were designed with the Primer Premier 5.0 software, and the housekeeping gene *β-actin* was used as an internal control for normalization (**Table 2**). The method 2^−ΔΔCt^ was used to calculate the levels of mRNA expression ratio (*R*) (Livak and Schmittgen, 2001).

**Table 2:** Primers used for qPCR amplification from Wuchang bream.

Target gene	Accession no.	Primer sequences (from 5′ to 3′)	Product length (bp)	Amplification efficiency (%)
*gh*	AF332563	F: TGTCGGTGGTGCTGGTT	212	105.32
		R: CGCCTCAATGGAGTCAGAGT		
*ghr1*	JN896373	F: AGCCTCCTCCTGAATCCT	186	95.42
		R: TTCCAGCAGTGAGAAGGTAT		
*ghr2*	JN896374	F: AGAGAATGTGAAGATAGGATGGA	216	107.15
		R: TAGGAATGAGAATGAAGATGGAGT		
*igf-1*	JQ398496	F: CCGATTTAAGGTCCGTATT	243	96.93
		R: GTGCAGCCGTAGTTCAGTT		
*cat*	KF378714	F: GTTTCCGTCCTTCATCCACTCT	190	92.66
		R: GACCAGTTTGAAAGTGTGCGAT		
*gpx1*	KF378713	F: CTTTTGTCCTTGAAGTATGTCC	114	102.46
		R: CTTGAGGAAGACGAAGAGAGGG		
*β-actin*	AY170122	F: ACCCACACCGTGCCCATCTA	152	108.48
		R: CGGACAATTTCTCTTTCGGCTG		

### Ultra-Pathological Analysis

Liver samples were prefixed in 2.5% glutaraldehyde solution with 1 mm^3^ size and then fixed in the cold 1% aqueous osmium tetroxide. After rinsing three times with phosphate buffer solution (0.1 M, PH 7.4), the samples were dehydrated with a graded ethanol series and embedded in Epon 812, which were further sliced on a LKB-V ultramicrotome (Nova, Sweden). Ultra-thin sections were stained with lead citrate and uranyl acetate and finally examined under a HITACHI, HT-7700 electron microscope.

To quantify hepatocellular damages by ammonia stress, a semiquantitative evaluation was carried out by classifying the extent of the observed damage into five categories and rating microscopic sections relative to each other: “-” absent; “±” weakly developed; “+” moderately developed; “++” strongly developed; “+++” very strongly developed (Strmac and Braunbeck, 2002).

### Calculations and Statistical Analyses

The weight gain (WG), feed conversion rate (FCR), and specific growth rate (SGR) were calculated based on all the surviving Wuchang bream at the end of the experiment using the following formula:

$$\begin{array}{lll} \mathrm{WG} & = & 100\times (W_{2}-W_{1})/W_{1}(g)\\ \mathrm{SGR} & = & 100\times (W_{2}-W_{1})/T\\ \mathrm{FCR} & = & T_{F}/T_{W}(g)\\ \mathrm{Mortality}(\%) & = & 100\times D/50 \end{array}$$

Where, W_1_, initial body weight (g); W_2_, final body weight (g); T_F_, total feed consumption (g); T, feeding days; T_W_, total weight gain (g); D, dead fishes per tank.

All data were analyzed using the SPSS 22.0 for the Windows (SPSS Inc., United States). The findings of growth performance were assessed via one-way ANOVE with the Sidak’s *post hoc* test. The findings of ammonia exposure were analyzed by two-way repeated measures ANOVA test with time and treated groups as the factors. If the assumption of sphericity was violated, a Greenhouse-Geisser was applied. Then one-way ANOVA was used to test differences from the control group at each time point and a Sidak *post hoc* test was used to test the effect of ammonia exposure compared to time 0. A *p*-value < 0.05 was considered to be statistically significant. All of the values were expressed as mean ± SE.

## Results

### Growth Parameters

As shown in **Table 3**, final body weight, WG and SGR in both SY group and combination groups were higher than those in control group (*p* < 0.001, *p* = 0.01 and *p* < 0.001 in SY group; *p* = 0.047, *p* = 0.032 and *p* = 0.030 in combination group, respectively), while no significant differences were detected in these parameters between TP group and the control group (*p* = 0.999, *p* = 1 and *p* = 1, respectively). Moreover, there were no significant differences in FCR between three treatment groups and the control group (*p* = 0.254 in SY group, *p* = 0.979 in TP group and *p* = 0.912 in combination group).

**Table 3:** Growth performance of Wuchang bream juveniles fed diets supplemented with selenium yeast, tea polyphenols and their combination for 60 days.

Item	Control	SY group	TP group	SY + TP group
Initial body weight (g)	3.33 ± 0.18	3.15 ± 0.15	3.28 ± 0.04	3.21 ± 0.23
Final body weight (g)	6.56 ± 0.04	7.31 ± 0.08^∗^	6.51 ± 0.09	6.86 ± 0.07^∗^
Weight gain (%)	98.16 ± 3.68	133.44 ± 1.86^∗^	98.49 ± 4.49	116.46 ± 3.15^∗^
Specific growth rate (%)	1.13 ± 0.04	1.41 ± 0.02^∗^	1.14 ± 0.02	1.28 ± 0.04^∗^
Feed conversion rate (%)	2.9 ± 0.16	2.35 ± 0.17	3.08 ± 0.10	2.66 ± 0.21
Survival rate (%)	100	100	100	100

*Values are means ± SE; asterisks indicate significant differences between treatment groups and control group (p < 0.05).*

### Growth Hormone and Gene Expression Analysis

**FIGURE 1:**
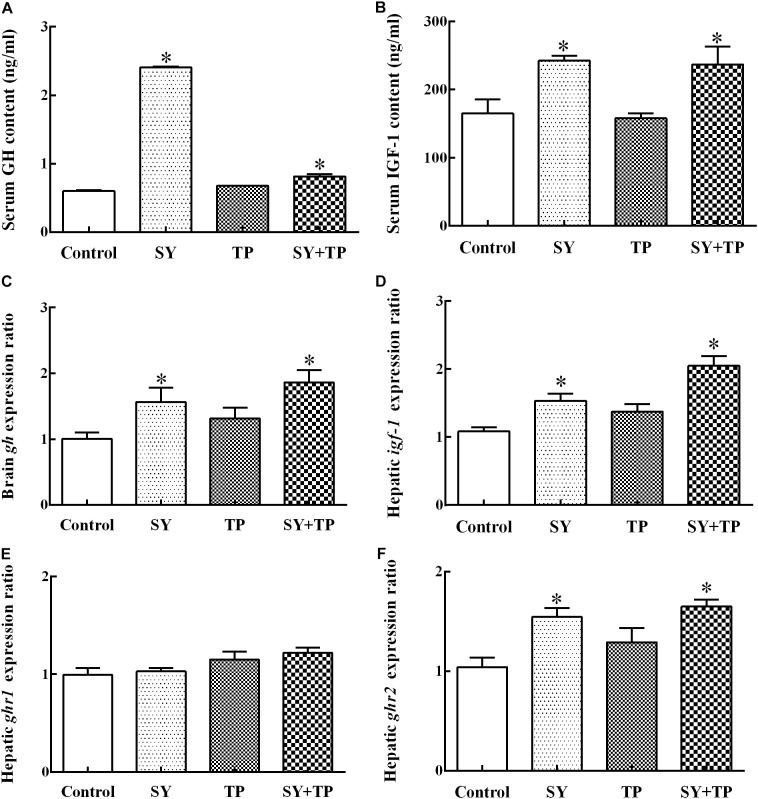
Effects of dietary selenium yeast and tea polyphenols on the content of GH and IGF-1 **(A,B)** and mRNA expression of *gh*, *ghr1*, *ghr2* and *igf-1* **(C,E,F,D)** in Wuchang bream after feeding trial. Data are expressed as means ± SE (*n* = 5). Asterisks indicate significant differences between treatment groups and the control group (*p* < 0.05).

After feeding trial, the contents of serum GH and IGF-1 in the control group were lower than those in the SY group (*p* < 0.001 and *p* = 0.011) and combination group (*p* < 0.001 and *p* = 0.017), whereas no significant differences were detected between the TP group and control group (*p* = 0.128 and *p* = 0.999) (**Figures 1A,B**). The mRNA expression levels of brain *gh* and hepatic *ghr2* as well as *igf-1* in the SY and combination groups were higher than those of the control group (*p* = 0.016, *p* < 0.001, and *p* = 0.037 in SY group, respectively; *p* ≤ 0.001 of the three parameters in combination group). In contrast, there were no significant differences in expression levels of *gh, ghr1, ghr2 and igf-1* between TP group and control group (*p* = 0.433, *p* = 0.879, *p* = 0.313, and *p* = 0.280, respectively) (**Figures 1C–F**).

### Oxidative Damage Parameters

**FIGURE 2:**
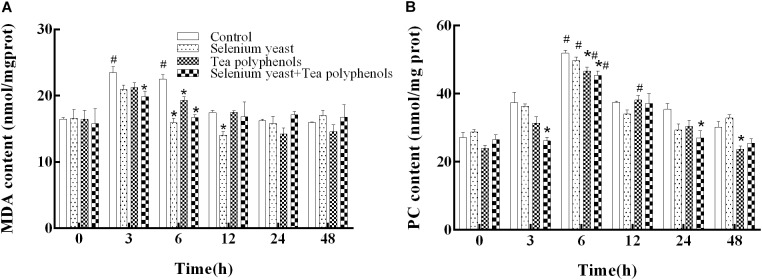
Effects of dietary selenium yeast and tea polyphenols on the contents of MDA **(A)** and PC **(B)** in the liver of Wuchang bream before and after acute ammonia exposure. Data expressed as means ± SE (*n* = 5). Asterisks indicate significant differences at the same time point between three treated groups and control group (*p* < 0.05). Hash symbols indicate significant differences at the same group between before and after ammonia stress (*p* < 0.05).

For hepatic LPO (measured as MDA), two-way repeated measures ANOVA showed a significant main effect of group (*p* = 0.003) and a significant effect of time (*p* < 0.001), suggesting that the increase of MDA after ammonia exposure was suppressed by the long-term dietary administration of SY and TP (**Figure 2A**). There was also a significant time × group interaction (*p* < 0.001). Compared with before ammonia exposure (0 h), higher levels of MDA were present only in the control group at 3 h and 6 h (*p* = 0.035 and *p* = 0.022, respectively), indicating the oxidative degradation of lipids induced by ammonia. Multiple comparison testing showed that MDA content of control group was higher than those in the SY group at both 6 h and 12 h (*p* ≤ 0.001 at the two time points), in TP group at 6 h (*p* = 0.017) and in the combination group at both 3 h and 6 h (*p* = 0.006 and *p* < 0.001).

For hepatic PC shown in **Figure 2B**, two-way repeated measures ANOVA showed a significant main effect of group (*p* = 0.003), a significant effect of time (*p* < 0.001) and a significant time × group interaction (*p* = 0.002). Compared with before ammonia exposure (0 h), hepatic PC levels of all experimental groups were significantly higher at 6 h after ammonia exposure (*p* = 0.034 in control group, *p* < 0.001 in SY group, *p* = 0.007 in TP group, and *p* = 0.018 in combination group), suggesting the occurrence of oxidative protein damage induced by ammonia. Multiple comparison testing showed that hepatic PC of control group was higher than those of the TP group at both 6 h and 48 h (*p* = 0.022 and *p* = 0.017), in the combination group at 3, 6, and 24 h (*p* = 0.005, *p* = 0.004 and *p* = 0.035, respectively), indicating that the increase of PC after ammonia exposure was inhibited by the long-term dietary administration of SY and TP.

### The Analysis of Antioxidant Enzyme Activity and Transcription

**FIGURE 3:**
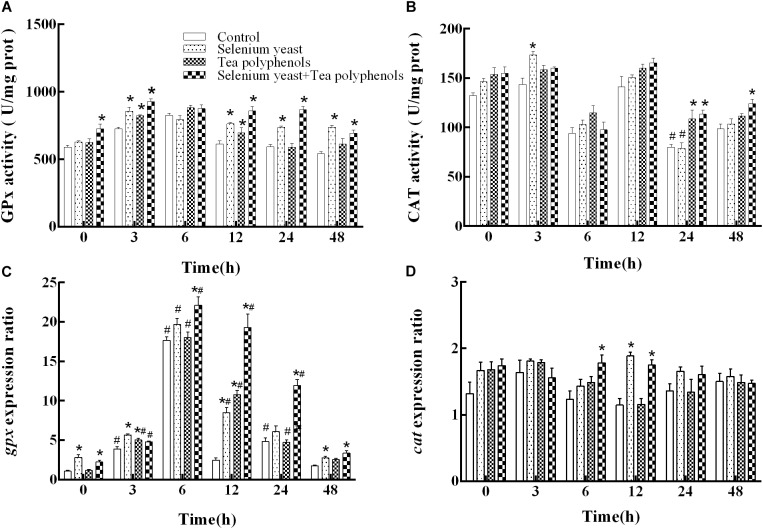
Effects of dietary selenium yeast and tea polyphenols on the activity and mRNA expression of antioxidant enzyme GPx **(A,C)** and CAT **(B,D)** in the liver of Wuchang bream before and after acute ammonia exposure. Data expressed as means ± SE (*n* = 5). Asterisks indicate significant differences between three treated groups and control group at the same time point (*p* < 0.05). Hash symbols indicate significant differences at the same group between before and after ammonia exposure (*p* < 0.05).

After ammonia exposure, the activity of GPx among all experimental groups presented an earlier increase and later decrease trend. Two-way repeated measures ANOVA showed a significant main effect of group (*p* < 0.001) and a significant time × group interaction (*p* < 0.001) (**Figure 3A**). Multiple comparison testing showed that GPx activity of control group was lower than those in SY group at 3, 12, 24, and 48 h (*p* = 0.003 at 3 h, *p* = 0.01 at 12 h, *p* < 0.001 at 24 and 48 h), in the TP group at 3 h and 12 h (*p* = 0.041 and *p* = 0.047) and in the combination group from 0 to 48 h except at 6 h (*p* = 0.005 at 0 h, *p* < 0.001 at 12, 24, and 48 h) (**Figure 3A**).

For *gpx* mRNA, two-way repeated measures ANOVA produced similar results that there were a significant main effect of group (*p* < 0.001), a significant effect of time (*p* = 0.025) and a significant time × group interaction (*p* < 0.001) (**Figure 3C**). Compared with before ammonia exposure (0 h), the mRNA levels of *gpx* were higher in control group at 3, 6, and 24 h (*p* = 0.005, *p* < 0.001, and *p* = 0.046, respectively), in the SY group at 6 h and 12 h (*p* = 0.002 and *p* = 0.011) and in the TP group from 3 to 24 h (*p ≤* 0.001 at 3, 6 and 12 h as well as *p* = 0.018 at 24 h) and in combination group from 3 to 24 h (*p* = 0.012, *p* = 0.006, *p* = 0.03, and *p* = 0.037, respectively). In the control group, the *gpx* gene expression was lower than those in SY group at 0, 3, 12, and 48 h (*p* < 0.001 at both 0 and 3 h, *p* = 0.002 at 12 h, and *p* = 0.033 at 48 h), in TP group at 3 and 12 h (*p* = 0.006 and *p* < 0.001) and in combination group from 0 to 48 h except at 3 h (*p* = 0.006 at 6 h and *p ≤* 0.001 at the other four time points).

For CAT, two-way repeated measures ANOVA test showed a significant main effect of group (*p* < 0.001) and a significant effect of time (*p* < 0.001) with a significant time × group interaction (*p* < 0.001) (**Figure 3B**). Compared with before ammonia exposure, the CAT activity was lower at 24 h in both the control group (*p* = 0.035) and the SY group (*p* = 0.019). In the control group, the CAT activity was lower than that in the SY group at 3 h (*p* = 0.002), in the TP group at 24 h (*p* = 0.019) and in the combination group at 24 h and 48 h (*p* = 0.036 and *p* = 0.009, respectively).

For the expression of *cat*, two-way repeated measures ANOVA test suggested a significant main effect of group (*p* = 0.009) and a significant time × group interaction (*p* = 0.003) (**Figure 3D**). Multiple comparison testing showed that the *cat* expression of control group were lower than those in the SY group at 12 h (*p* < 0.001) and in the combination group at the both 6 and 12 h (*p* = 0.014 and *p* = 0.001, respectively).

### Ultrastructural Observation

**FIGURE 4:**
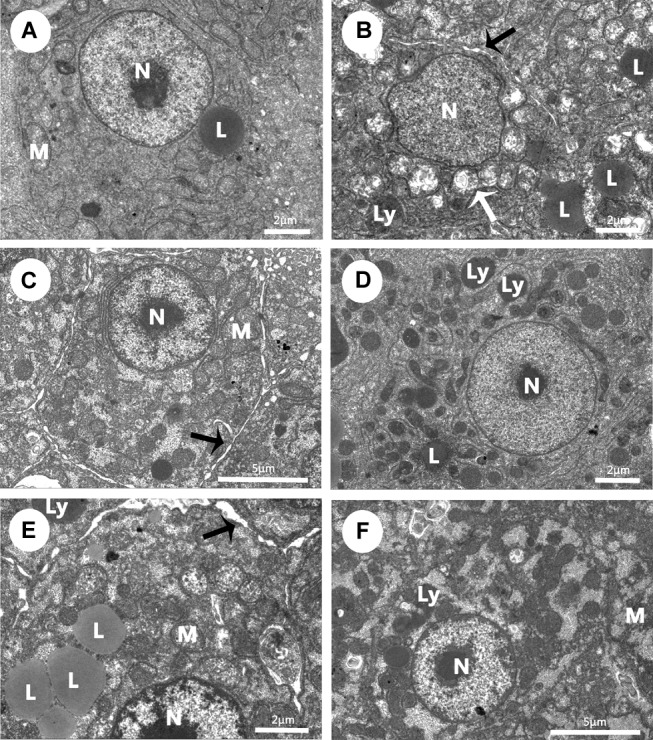
Effects of dietary selenium yeast, tea polyphenols and their combination on liver ultrastructure in Wuchang bream before and after acute ammonia exposure. **(A–C)** The liver of control group at 0 h before ammonia exposure **(A)** and 24 h and 48 h after ammonia exposure **(B,C)**. **(D)** The liver of SY group at 24 h after ammonia exposure. **(E)** The liver of TP group at 24 h after ammonia exposure. **(F)** The liver of the combination group at 24 h after ammonia exposure. White arrow: swollen mitochondria; black arrow: the dilation of intercellular space. Other *abbreviations* are for: L, lipid droplets; Ly, lysosomes; M, mitochondria; N, nuclei.

A semiquantitative evaluation of ultrastructural alterations in the liver of Wuchang bream before and after acute ammonia exposure is given in **Table 4**. Before ammonia exposure, liver ultrastructure in all experimental groups showed a normal appearance with a centrally located nucleus, numerous mitochondria and occasional lipid droplet (**Figure 4A**). After ammonia exposure, the liver damage of control fish increased with the exposure time within 24 h and was characterized with swollen mitochondria, the increase of lipid droplets, the deformation of nuclear envelope as well as the dilation of intercellular space (**Figure 4B**). In contrast, the liver of the SY and combination groups only showed minor increases of lysosomes and lipid droplets within 24 h after ammonia exposure (**Figures 4D,F**), whereas hepatic mitochondria of TP group became slightly swollen and a large number of different-sized lipid droplets accumulated in the hepatocyte along with widening of intercellular spaces (**Figure 4E**). At 48 h, the liver of three treatment groups showed great recovery while the dilation of intercellular space was still in control group (**Figure 4C**).

**Table 4:** Semiquantitative evaluation of ultrastructural alterations in the liver of Wuchang bream before and after acute ammonia exposure.

		Time					
Groups		0 h	3 h	6 h	12 h	24 h	48 h
Control	Swollen mitochondria	−^a^	±	+	++	+++	−
	Amount of lysosomes	±	±	±	+	+	±
	Amount of lipid droplets	±	+	++	++	++	+
	Deformation of nuclear envelope	−	−	−	±	+	−
	Dilation of intercellular space	−	−	±	±	±	±
SY	Swollen mitochondria	−	±	±	±	±	−
	Amount of lysosomes	±	±	±	±	+	±
	Amount of lipid droplets	±	±	+	+	++	+
	Deformation of nuclear envelope	−	−	−	−	−	−
	Dilation of intercellular space	−	−	−	−	−	−
TP	Swollen mitochondria	−	−	−	−	±	−
	Amount of lysosomes	±	±	±	±	±	±
	Amount of lipid droplets	±	±	±	++	++	+
	Deformation of nuclear envelope	−	−	−	−	−	−
	Dilation of intercellular space	−	−	±	±	±	−
SY + TP	Swollen mitochondria	−	−	−	±	±	−
	Amount of lysosomes	±	±	±	±	±	±
	Amount of lipid droplets	±	±	±	±	+	±
	Deformation of nuclear envelope	−	−	−	−	−	−
	Dilation of intercellular space	−	−	−	−	−	−

*^a^ −, absent; ±, weakly developed; +, moderately developed; ++, strongly developed; +++, very strongly developed.*

## Discussion

### Effects of Selenium Yeast and Tea Polyphenols on Growth Performance

In the present study, juvenile Wuchang bream both in the SY and combination groups showed significant increases on final weight, WG and SGR, indicating that single SY supplementation as well as the combined use of SY and TP promoted feed intake, feed conversion and growth. In agreement with our results, growth improvement have been found in hybrid striped bass (*Morone chrysops* × *Morone saxatilis*), rainbow trout (*Oncorhynchus mykiss*) and marron (*Cherax cainii*) fed diets supplemented with SY (Cotter et al., 2008; Rider et al., 2009; Nugroho and Fotedar, 2013). It is well known that microelement selenium is very critical for normal animal growth and development (Thomson, 2004). Organic selenomethionine, as the main components of SY, can be metabolized, stored as selenoprotein and finally taken part into pathways of protein mechanism (Schrauzer, 2006), which help to improve fish growth performance (Lin and Shiau, 2005; Han et al., 2011). In this regard, growth promotion of fish in the SY and combination groups in our study might be involved with nutritional function of selenium. However, the differences in growth parameters were not significant between the TP and control groups. Similarly, several studies reported that supplemental TP in diets had no effects on growth parameters like WG and SGR in *Paralichthys olivaceus* and *O. mykiss* (Cho et al., 2007; Xu et al., 2008). Since there was no additive effects in combination group compared to SY group, the growth-promoting effect in the combination group might be due to SY alone.

The GH/IGF-1 axis plays a crucial part in the regulation of fish growth (Reinecke et al., 2005; Berryman et al., 2008). GH is secreted from the pituitary, circulates through the blood to the liver and then binds to a membrane-bound growth hormone receptor (GHR) (Zhu et al., 2001; Saera-Vila et al., 2005), which further stimulate the synthesis and secretion of hepatic IGF-1. IGF-1 then acts on target cells to stimulate cell proliferation and differentiation and eventually body growth (Reindl and Sheridan, 2012). Previous studies have proved that endogenous and exogenous GH can promote the growth of fish (Johnsson and Björnsson, 1994; Perez-Sanchez and Le Bail, 1999; Norbeck et al., 2007). It has been demonstrated that IGF-1 protein and mRNA levels in fish liver were significantly positively correlated with body weight gain and SGR in Nile tilapia (Uchida et al., 2003; Vera et al., 2006). In the current study, we for the first time observed significant increases of GH and IGF-1 at protein and transcriptional levels as well as the upregulation of *ghr2* mRNA in the SY and combination groups, however, the administration of TP alone didn’t have significant effects on the contents and mRNA expression of serum GH and IGF-1. This suggested that SY alone might improve fish growth through regulating GH/IGF-1 axis. Further marked upregulation of *ghr2* gene expression in both SY group and the combination group revealed that the role of GH was mediated primarily by GHR2 in our study. Khan et al. (2016) reported that diets supplemented with selenium nanoparticles for 70 days increased significantly serum GH levels in juvenile mahseer (*Tor putitora*) and higher GH levels led to enhanced growth. Also, Collipp et al. (1984) found a significant relationship between GH level and selenium nutritional status and confirmed that selenium supplementation influenced significantly serum GH level and the subsequent growth performance of fish. On the other hand, the Study of Köhrle and Gärtner (2009) also demonstrated that selenium affected secretion of thyroid hormones, since selenium is a vital component of the deiodinase enzyme, which is necessary for the transformation of tetraiodothyronine (T3) to the biologically more active triiodothyronine (T4). And thyroid hormone T3 could increase the levels of GH mRNA in the pituitary cells of teleost fish (Moav and McKeown, 1992; Farchi-Pisanty et al., 1995). Taken together, growth performance improvement in the SY and combination groups might be derived from SY supplement and associated with nutritional function of selenium and upregulated responses of endocrine GH/IGF-1 axis.

### Effects of Selenium Yeast and Tea Polyphenols on Ammonia Tolerance

Oxidant/antioxidant status in fish is commonly used to assess whether the liver is function normally or injured or diseased (Velmurugan et al., 2007). MDA is a product of LPO, which has a strong biological toxicity and can damage cell structure and function (Sies, 1991; Vaughan, 1997). PC is a type of protein oxidation mainly induced by ROS, thus the assay for PC can quantify oxidative damage to proteins (Stadtman and Levine, 2000; Suzuki et al., 2010). In the present study, hepatic MDA and PC in all experimental groups significantly increased compared with before ammonia, which suggested that acute ammonia exposure induced oxidative stress and protein damage in the liver of juvenile Wuchang bream. Elevated MDA levels have been reported in bighead carp *Hypophthalmythys nobilis* larvae (Sun et al., 2012), sea bass (*Dicentrarchus labrax*) (Sinha et al., 2015), yellow catfish (*Pelteobagrus fulvidraco*) (Zhang et al., 2016) after exposure to ammonia and indicated the occurrence of oxidative stress in fish body. Significantly increased PC were also detected in the brain of mudskipper (*Boleophthalmus boddarti*) exposed to a sublethal concentration of NH_4_Cl (8 mmol/L) for 48 h (Ching et al., 2009). In the present study, it should be noted that levels of MDA and PC in three treated groups were persistently lower than those of control group after ammonia exposure, which implied that dietary SY and TP can alleviate oxidative stress and damage caused by ammonia to some extent. Küçükbay et al. (2009) reported that MDA concentrations in the serum and muscle of rainbow trout decreased linearly with increasing sodium selenite or selenomethionine supplementation. Nootash et al. (2013) found that green tea supplement at the dose of 100 mg kg^−1^ could decrease MDA contents in the serum of rainbow trout.

Catalase and GPx are two main antioxidant enzymes, which can scavenge excessive ROS and reduce the damage by LPO and PC (Nordberg and Arner, 2001). The detections of CAT and GPx levels are usually considered as indicators of antioxidant status in fish. In the present study, compared with before ammonia, the expression of *gpx* among all groups showed a trend that increased first and then decreased, indicating an activation of antioxidant response in the liver of Wuchang bream after ammonia exposure. Previous studies found that ammonia stress could change the gene expressions of hepatic antioxidant enzymes like CAT and GPx to protect fish from oxidative stress by ammonia (Hegazi et al., 2010; Cheng et al., 2015). Sinha et al. (2014) also reported that GPx activity in the liver of goldfish increased significantly at 48 h and then recovered at 180 h after ammonia exposure. Furthermore, the enzymatic activity and transcription of hepatic CAT and GPx were higher in three treatment groups than those of the control group whenever before and after ammonia exposure, which suggested stronger antioxidant capacity in the treated groups. In fact, one of the important biological functions of selenium is its antioxidant effects, since it forms selenocysteine, which is the part of the active center of GPx (Watanabe et al., 1997). Rider et al. (2009) reported that a diet supplemented with SY increased GPX activity in the liver of rainbow trout. Lin and Shiau (2005) also showed that hepatic GPx activity were enhanced significantly in the juvenile grouper (*Epinephelus malabaricus*) fed diets with selenomethionine. As another natural antioxidant, TP are well known with their antioxidant and immune function (Xu et al., 2007). Lin et al. (1998) demonstrated that a diet supplied with 2.5% green tea leaves induced significantly hepatic CAT activity in rats. Supplementation of 0.2% green TPs in drinking water of hairless mice for 30 days significantly increased the activity of CAT and GPx in small intestine, liver, and lung compared with controls (Agarwal et al., 1993). Unfortunately, it is very limited or complete lack of information on the effects of TP on the antioxidant system in fish. On base of above, our finding suggested dietary SY and TP can enhance antioxidant capacity by inducing CAT and GPx and resist oxidative stress and damage by ammonia. In the present study, the recovery of MDA and PC in three treatment groups at the end of ammonia exposure experiment further confirmed that the excessive ROS and protein damages were effectively scavenged by CAT and GPx. Further, higher levels of hepatic GPx and CAT as well as *gpx* mRNA for the combination group at 48 h after ammonia exposure indicated that the combination of SY and TP is more effective on antioxidant capacity than single supplementation of either SY or TP.

It is reported that the liver is one of the targeted organs of ammonia. Previous investigators have documented that ammonia nitrogen can cause liver histopathological changes including vacuolization of hepatocytes, fatty deposition, dilation of the sinusoids and capillary congestion (Ravindrababu and Neeraja, 2012; Rodrigues et al., 2014; Da Cruz Ribeiro et al., 2017). Ultrastructural observation showed that ammonia exposure induced altered nuclei, the increase of primary and secondary lysosomes as well as lipid droplets accumulation in the liver of the *Siganus rivulatus* (Abdelmeguid et al., 2013). In our study, the liver ultrastructure of control fish underwent irregular nuclear, swollen mitochondria and dilation of intercellular space under acute ammonia stress, which was similar with the previous studies (Ravindrababu and Neeraja, 2012; Rodrigues et al., 2014; Da Cruz Ribeiro et al., 2017). Meanwhile, we only observed increased lysosomes in the SY and combination groups and slight swollen mitochondria as well as dilation of intercellular space in TP group. Apparently, ultrastructural changes in liver damage induced by acute ammonia exposure were much more severe in control group than in three treated groups. It is clearly that our pathological findings provide direct insights that dietary SY and TP can relieve hepatocytes damage and make contributes to the recovery of liver structure in fish after acute ammonia exposure. As the mentioned in the present experimental results, hepatic ultrastructural recovery in three treated groups may be more likely due to elevated antioxidant responses and detoxification.

## Conclusion

The current results in this study suggested that basal diets supplemented with SY could effectively promote growth of Wuchang bream juveniles by regulating GH/IGF axis. Moreover, single SY and TP supplementation or their combination could enhance resistance to ammonia stress by improving the activity and transcription of antioxidant enzymes and reducing MDA and PC levels. Furthermore, the combination use of SY and TP in fish feed have better effects than the single SY and TP supplementation in term of antioxidant responses and histological recovery.

## Author Contributions

HG and LL: concept and design of the experiments and wrote the manuscript. WL, JH, LW, DZ, and XW: conduction of the lab experiments and data analysis. HG and DL: data collection and handling.

## Conflict of Interest Statement

The authors declare that the research was conducted in the absence of any commercial or financial relationships that could be construed as a potential conflict of interest.
